# Healthy together Victoria and childhood obesity—a methodology for measuring changes in childhood obesity in response to a community-based, whole of system cluster randomized control trial

**DOI:** 10.1186/s13690-016-0127-y

**Published:** 2016-04-25

**Authors:** Claudia Strugnell, Lynne Millar, Andrew Churchill, Felice Jacka, Colin Bell, Mary Malakellis, Boyd Swinburn, Steve Allender

**Affiliations:** World Health Organization’s Collaborating Centre for Obesity Prevention, Deakin Population Health, Deakin University, Geelong, Australia; School of Health and Social Development, Deakin University, Melbourne, Australia; Formerly Department of Health & Human Services, Population Health and Prevention Strategy, ᅟ, VIC Australia; IMPACT Strategic Research Centre, Deakin University, Geelong, Australia; Department of Psychiatry, The University of Melbourne, Melbourne, Australia; Centre for Adolescent Health, Murdoch Children’s Research Institute, Melbourne, Australia; Black Dog Institute, Sydney, Australia; School of Medicine, Deakin University, Geelong, Australia; School of Population Health, The University of Auckland, Auckland, New Zealand

**Keywords:** Evaluation, Systems, Community-based interventions

## Abstract

**Background:**

Healthy Together Victoria (HTV) - a complex ‘whole of system’ intervention, including an embedded cluster randomized control trial, to reduce chronic disease by addressing risk factors (physical inactivity, poor diet quality, smoking and harmful alcohol use) among children and adults in selected communities in Victoria, Australia (Healthy Together Communities).

**Objectives:**

To describe the methodology for: 1) assessing changes in the prevalence of measured childhood obesity and associated risks between primary and secondary school students in HTV communities, compared with comparison communities; and 2) assessing community-level system changes that influence childhood obesity in HTC and comparison communities.

**Methods:**

Twenty-four geographically bounded areas were randomized to either prevention or comparison (2012). A repeat cross-sectional study utilising opt-out consent will collect objectively measured height, weight, waist and self-reported behavioral data among primary [Grade 4 (aged 9-10y) and Grade 6 (aged 11-12y)] and secondary [Grade 8 (aged 13-14y) and Grade 10 (aged 15-16y)] school students (2014 to 2018). Relationships between measured childhood obesity and system causes, as defined in the Foresight obesity systems map, will be assessed using a range of routine and customised data.

**Conclusion:**

This research methodology describes the beginnings of a state-wide childhood obesity monitoring system that can evolve to regularly inform progress on reducing obesity, and situate these changes in the context of broader community-level system change.

## Background

Childhood obesity immediately and distally impacts physical and psychological health [1, 2]. The stubbornly high prevalence of obesity globally [3], and persistence of childhood obesity into adulthood [2, 4], with resultant increased morbidity and mortality [5], highlights the imperative to develop effective prevention and monitoring strategies for childhood obesity.

Evidence [6–8] of success in preventing childhood obesity [6, 7] in a variety of specific settings (e.g., school-based [8–10], community-based [11], home-based [12],) is tempered by challenges in sustaining the long-term impact of these interventions and applying the interventions at the population level [6]. The most recent Cochrane review of obesity prevention [6] has identified that multifaceted and multilevel strategies are required to prevent obesity. The Foresight Obesity Systems Map [13] provides corroboration for this assessment through the visualization of obesity as a complex systems problem [14]. Support is growing for population level efforts to prevent childhood obesity to apply systems thinking [15] in the expectation that systems thinking may improve intervention implementation, effectiveness and sustainability of changes. To date, no known initiative has applied a ‘whole of systems’ approach to prevent childhood obesity [16]. Healthy Together Victoria’s (HTV) large-scale complex whole of system approach to the primary prevention of chronic disease, in the state of Victoria, Australia is a world first. Taking a population-level approach to reducing chronic disease and specifically obesity through improving associated determinants (physical inactivity, poor diet quality, smoking and harmful alcohol use) among children and adults in the specific communities where they “live, learn, work and play” [17]. Due to the broad scope and the adaptive nature of HTV, detailed methods are beyond the remit of this article but can be found at http://www.healthytogether.vic.gov.au/resources/index.

Commencing in 2012, HTV’s multi-faceted intervention includes a boosted capacity at the local level of >170 staff in 12 communities [18]. These personnel were employed to support and deliver ‘system activation’ for healthy environments and healthy living in schools, early childhood settings, workplaces and communities through a variety of means. Systems activation refers to initiating actions on the systems that influence the health and well-being of individuals, families and communities. These actions include delivering multiple strategies, policies and initiatives at both the state and local levels to target all Victorians. Complementing the state-wide systems activation and programs, HTV also contributes resources and effort towards improving the health of children through a quality framework to support the creation of healthier school environments through a range of services such as the Healthy Together Victoria Achievement Program and Healthy Eating Advisory Service and is complemented by a range of community based healthy living programs and state-wide and local social marketing of health promotion messages.

HTV includes a cluster randomized trial of the ‘whole-of-system’ intervention. With increasing intervention complexity, evaluation approaches require a degree of flexibility in order to accordingly engage with complexity. In this paper, we outline the methodological approaches that will be taken to comprehensively measure the impacts of HTV on childhood obesity through two parallel research programs with the following aims:

1. To measure the impacts of HTV on anthropometry and obesogenic behaviors among Victorian children and the environments in which they live, learn and play
2. To measure the effects of change to community-level system factors on rates of childhood obesity among Victorian children.

## Methods/design

### Setting

Healthy Together Victoria, an initiative of the State Government of Victoria, was initially funded in partnership with the Commonwealth Government of Australia [17]. Victoria is a state of approximately 5.8 million people in the South-Eastern region of Australia [19]. As described above, the centrepiece of HTV is a funded workforce of approximately 170 people across the state in local government, community health and non-government organisations whom take a range of actions at the community and state level [18]. These actions are varied but can include: policy and leadership for health promotion; providing quality improvement frameworks for early childhood services, schools and workplaces; supporting policy development/implementation and targeted strategies within settings; working with food growers, producers and sellers to increase access to healthy food, especially disadvantaged populations; working with urban planning to support active transport; working with sport and recreation centres to increase healthy food access; building leadership for prevention; and social marketing of health promotion messaging www.healthytogether.vic.gov.au [18]. The cluster randomized trial, embedded within the wider HTV initiative, includes 12 prevention areas spread across 14 local government authority (LGAs) (Healthy Together Communities) and 11-matched comparison areas spread across 12 LGA’s in Victoria, Australia (with one comparison community acting as a matched comparator for two prevention areas). Matched randomisation of clusters was based on socio-demographic indices [Socioeconomic Index for Areas (SEIFA)] [20] and chronic disease risk factor prevalence (i.e., unhealthy weight)] of adults within participating LGA’s and was conducted by the Department of Health and Human Services (Victoria). The wider HTV evaluation has a multitude of components, several of which are outlined in Table 2. The methodology described here captures (1) how the core primary and secondary outcomes will be measured and (2) how these relate to community-level system change.

### Research plan aim 1: to measure the impacts of HTV on anthropometry and obesogenic behaviors among Victorian children and the environments in which they live, learn, and play

Under aim one it is hypothesized that:

1. School children in intervention LGAs compared with those in comparison LGAs will record relative decreases in BMI z-scores of at least 0.18 over the measurement period,
2. The prevalence of overweight/obesity among school children in intervention LGAs will decrease over the intervention period, compared to those in the comparison LGAs, and
3. The levels of physical activity will increase, sedentary behavior will decrease and diet quality will improve over the intervention period among school children in the intervention LGAs compared with those in the comparison LGAs.

### Study design, sampling and sample size

Standard approaches for sample size calculations within cluster randomized trials were made (in GenStat V14.2) based on the comparison of Body Mass Index standardized for age and sex (BMI-z) [21] scores between intervention (12 clusters) and comparison (12 clusters) communities using power of 0.8 and 0.05 for significance test. The clustered trial involves intervention at the area level and thus an intra-class correlation of 0.1 for the BMI-z was used, based on previous school-based research [22–24]. Baseline BMI-z (0.65) and standard deviation (0.93) were taken from our previous studies of >1800 [22] school children and the expected change in BMI-z over the 4 years was set to 0.18. The justification for this estimated change over 4 years of 0.18 is based on the findings from our previous interventions within the same age groups (primary [22] and secondary students [24]) and was associated with a 3 percentage point reduction in overweight and obesity in these populations.

Using the parameters above, the sample size calculations indicated a need for ≥ 25 children in each of the 4 year levels (Grade 4, Grade 6, Grade 8 & Grade 10) to be assessed in each intervention/comparison area to determine significant differences in BMI-z score between intervention and comparison communities. A repeat cross-sectional design will assess all participating students in each grade level per intervention and comparison community and repeated samples of students on a biennial basis in 2014, 2016 and 2018 during Term 3 (Jul - Sept) of the Victorian School Term (giving a total of approximately >7,200 children assessed once).

### School and participant recruitment

A list of all Victorian schools will be stratified by LGA and a random sampling technique with replacement used to invite three primary and three secondary schools within each of the prevention and control areas (spanning 26 LGAs). For example, should all schools that have been selected at random decline to participate, then the next three schools selected at random will be invited. Written invitations will be sent to the school Principal with follow-up phone calls, emails and/or visits used to confirm participation. All students in Grade 4 and Grade 6, or Grade 8 and Grade 10, will be invited to voluntarily participate through the distribution of the plain language statement and opt-out consent form. An opt-out recruitment procedure will be used, whereby participants are only required to return a signed opt-out form if they or their parents/guardians do not want their child to participate. However, if an individual child does not wish to be measured or surveyed they do not have to participate, regardless of having a signed opt-out consent form. This study has been approved by Deakin University’s Human Research Ethics Committee (2013-095), the Victorian Department of Education and Training (2013_002013) and the Catholic Archdiocese of Melbourne, Sandhurst, Ballarat and Sale.

### Study protocol and procedures

Approximately 10 weeks are allocated for the recruitment of schools and participants in Term 2 (April–June) and 10 weeks for the collection of data during Term 3 in each study year. Consenting participants will be invited to complete a self-report questionnaire and have their height, weight and waist circumference measured (see Table 1). All measurements will be conducted throughout the school day by trained research staff using calibrated equipment and is expected to take between 20–30 min to complete the questionnaire and 3–5 min to complete anthropometric measures.

**Table 1:** Primary and secondary outcomes of interests and proposed instrument/measure

Item	Outcome(s) of interest	Proposed Instrument/measure
Anthropometry	• Change in BMI-z score • Change in overweight and obesity prevalence • Change in abdominal obesity prevalence	Height, weight & waist circumference
Physical activity and Sedentary behavior	• Change in minutes per day (mins.d^−1^) spent in daily moderate-to-vigorous physical activity (MVPA) and sedentary time • Change in the proportion of participants meeting Australia’s physical activity and sedentary behavior guidelines for children (5–12 years) [33] and young people (13–17 years) [34] • Change in levels of perceived psychosocial influences on physical activity participation	Modified questionnaire containing items from the Core Indicators and Measures of Youth Health [30] and SHAPES [28] surveys.
Duration, intensity and perceived psychosocial influences		
Diet	• Change in typical/usual serves of fruit and vegetable daily • Change in typical/usual serves of non-core foods • Change in typical/usual serves of sugar-sweetened beverages • Change in the proportion of participants meeting the Australian Dietary guidelines for fruit and vegetable intakes [38] • Change in usual caffeinated energy drink intake	Modified version of the Simple Dietary Questionnaire (Parletta N, Frensham L, Peters J , O’Dea K, Itsiopoulos C. Validation of a Simple Dietary Questionnaire with adolescents in an Australian population, unpublished). [56]
Type, frequency		
Moods and Feelings	• Change in depressive symptom score	Grade 8–10: Short Moods and Feelings Questionnaire [47]
Emotional wellbeing		
Quality of Life	• Change in the global score • Change in psychosocial health summary score • Change in physical health summary score	Paediatric Quality of Life Inventory (PedsQL) [40]
Environments	• Change in school policy environment • Change in school physical environment • Change in school economic environment • Change in school socio-cultural environment	Primary Schools: Be Active Eat Well Environment questionnaire Secondary schools: It’s Your Move Environment Questionnaire

### Measures

#### Primary outcome

##### Anthropometry: height weight and waist circumference

Height will be measured to the nearest 0.1 cm using a portable stadiometer (Charder HM-200P Portstad, Charder Electronic Co Ltd, Taichung City, Taiwan) and weight to the nearest 0.1 kg using an electronic weight scale (A&D Precision Scale UC-321; A7D Medical, San Jose, CA) without shoes and whilst wearing a light layer of clothing (e.g., shirt/t-shirt and shorts/long pants/skirt). Age and sex-specific body mass index (BMI) z-scores will be calculated using the World Health Organizations’ growth reference to categorize weight status [21]. Waist circumference will be taken over light clothing using the cross-over technique at the midway point between the lower costal border (10^th^ rib) and the top of the iliac crest, in the mid-axillary line, perpendicular to the long axis of the trunk [25] using a steel tape measure (Lufkin W606PM) to the nearest 0.1 cm. Waist circumference data will be used to examine abdominal obesity when mean waist circumference is greater than the 90th percentile [26] according to the International Diabetes Federation using the British age and sex-specific growth reference [27]. Two measurements will be taken for height, weight and waist and the average calculated unless a third measure be needed when a discrepancy of 0.5 cm for height and waist and 0.5 kg for weight occurs.

#### Secondary outcomes

##### Physical activity and sedentary behavior: questionnaire

After evaluating current tools to examine self-reported physical activity and sedentary behavior among youth, the School Health Action, Planning and Evaluation System (SHAPES) questionnaire was identified as an exemplar as it had previously demonstrated moderate construct validity correlations (accelerometry) for average Moderate-to-Vigorous Physical Activity (MVPA) min.d^−1^ (*r* = 0.44, *P* < 0.01) among Grade 4 to Grade 10 Canadian children [28]. This construct validity correlation is considerable given that a recent systematic review of physical activity questionnaires found median criterion validity correlations to be *r* = 0.30 to 0.39 for new questionnaires and *r* = 0.25 to 0.41 for existing questionnaires [29]. The authors of the SHAPES questionnaire were contacted and they highlighted success towards uniform/standard measurement of physical activity and sedentary behavior across Canada (in consultation with several other universities) [30]. The use of standard measures potentially enables comparisons with other studies. Therefore, three question items from the standardised Canadian Core Indicators and Measures of Youth Health – Physical Activity & Sedentary Behavior Module [30] questionnaire examining duration spent in MVPA, sedentary behavior and mode of active transport to and from school were also included alongside 15 question items from the SHAPES questionnaire examining active transport, frequency of physical education and perceived psychosocial influences on physical activity (social support and parental modelling) [28]. The SHAPES questionnaire and Core Indicators and Measures were developed in Canada and based on the Canadian physical activity [31] and sedentary behavior [32] guidelines for youth. These guidelines are identical to the Australian physical activity and sedentary behavior guidelines in recommending ≥ 60 min/day of MVPA and ≤ 2 h/day of electronic media for entertainment [33, 34]. This supports the use of these question items in the Australian context as they are likely to have similar psychometric properties among Australian youth. Through these questionnaires, duration spent in MVPA (in total) and sedentary behavior (outside of school) over the previous 7 days will be recalled by participants and adherence to Australia’s physical activity and sedentary behavior guidelines for children (5–12 years) [33] and young people (13–17 years) [34] calculated. Contextual information relating to participation (e.g., number of television units in house and active transportation) and perceived psychosocial influences on physical activity participation (e.g., social support, parental modelling) will also examined.

### Diet quality: questionnaire

Self-reported usual dietary intake is commonly examined through food frequency questionnaires at the population level as they are quick to complete, inexpensive and have low participant burden in large scale studies [35]. The advantage of a food frequency questionnaire compared to a 24 h diet recall questionnaire is that usual intake can be estimated, whereas a singular 24 h diet recall is subject to intra-individual variation and requires several administrations to capture usual intake, which subsequently increases costs and subject burden [35]. The Simple Dietary Questionnaire (SDQ) was selected to examine dietary behaviors as it has been validated among Australian school children aged 13 to 16 years and demonstrated moderate validity correlations (*r* = 0.42 to 0.57) for fruit and vegetable intake with 24 h diet recall (Parletta N, Frensham L, Peters J , O’Dea K, Itsiopoulos C. Validation of a Simple Dietary Questionnaire with adolescents in an Australian population, unpublished). In comparison, a recent systematic review of food frequency questionnaires among youth found validity correlations ranged from *r* = 0.1 to 0.8 [36], further supporting the use of the SDQ in the current study. The SDQ is a quick to complete 27-items questionnaire that examines intakes of fruit and vegetables, dairy (milk, cheese, ice-cream), sweetened beverages and non-core foods (e.g., takeaway food and candy) and was based on the former (2003) Australian dietary guidelines [37]. The number of fruit and vegetable serves usually eaten each day will be specifically examined to detect changes in the proportions of participants adhering to the current Australian Dietary Guidelines for daily serves of fruit and vegetables and the questionnaire was amended to allow for half-serves to be recorded for fruit and vegetable consumption (e.g., 1 serve, 1.5 serves, 2 serves… etc.) [38]. In addition, intakes of core and non-core foods and beverages will be examined for changes over time.

### Quality of life: questionnaire

Given that childhood obesity has both physical and psychological health consequences [1, 2], the assessment of health-related quality is important to capture the multidimensional state of health [39]. Although quality of life is a complex concept, a critical search of the literature supported the use of the Paediatric Quality of Life Inventory 4.0 (PedsQL)™ [40] to examine perceived health-related quality of life among participants. The PedsQL has been widely used in Australian [41–44] and international (>350 citations in Medline) studies of children and adolescents to examine health-related quality of life in a variety of settings, thus enabling accurate comparison to other studies due to the application of the same instrument. It is a quick to complete (1-page) 23-item questionnaire that examines self-rated physical health, emotional health, school functioning and social health. It has been subject to extensive psychometric testing in a variety of languages [45] with high internal consistency observed among children 8 to 11 years (α = 0.90–0.91) in the United States and validity through comparison with children with diagnosed chronic health conditions (effect size *d* = 0.63–0.72) [40]. The questionnaire includes four domains (physical health, emotional health, school functioning and social health) that will be examined individually and in combination both cross-sectionally and change over time. The scores are summed to provide an overall global health-related quality of life score as well as generating physical and psychosocial sub-scale scores.

### Depressive symptomology: questionnaire (Grade 8 and Grade 10)

Given the relationship between obesogenic risk factors (physical activity, sedentary behavior and diet) and depressive symptoms among adolescents [46], this important outcome will be examined. The Short Mood and Feelings Questionnaire (SMFQ) [47] has previously demonstrated high internal consistency among Australian children aged 10–14 years (α > 0.85) [48], with the additional benefit of having international application and validation among children and adolescents [47]. The SMFQ was selected to examine depressive symptoms as clinical diagnosis of depression through clinical psychiatric interviews is impractical for population based-studies [47]. Due to the nature of the questionnaire items, it was felt that the questionnaire be administered to secondary school students only (Grade 8 and Grade 10), despite being psychometrically tested among Australian children as young as 10 years of age [48]. The responses from the questionnaire will be collated into a SMFQ score, which will be used as a measure of depressive symptomology.

#### Demographic characteristics

Demographic characteristics of participants will be captured through the self-report questionnaire and include: date of birth, gender, residential postcode, language spoken at home, country of birth and ancestry. These characteristics will be used primarily as covariates for the primary and secondary outcome measures.

#### School environments

Within each participating school, approximately 1–3 members of staff will be invited to complete a school environment audit (Primary Schools: Be Active Eat Well Environment questionnaire Secondary schools: It’s Your Move Environment Questionnaire). This survey, examines the schools’ policies and environments in relation to physical activity and healthy eating (e.g., number and type of physical activity and healthy eating policies; types of unhealthy food available in school canteens and distance of nearest takeaway shop, frequency and duration of physical education and sport education). This survey takes approximately 15 min to complete and will be conducted during the school day (typically at recess or lunch) [49]. These questionnaires were developed for two previous community-based interventions and were examined for face validity by the study’s executive but these instruments have not been subject to formal psychometric testing due to the nature of the question items. School policy, physical, economic and socio-cultural environments will be examined for change over time and their relationship with obesity and associated risk factors.

### Data analysis

Cross-sectional (within year) and longitudinal (across region/LGA) multilevel analyses will be conducted to consider different exposure variables (condition) and the effects of clustering (within school, between LGA) and other potential confounders (age, gender, socioeconomic position). A multilevel growth model [50] will be used to examine how BMI z-score, and other dependent variables (physical activity, sedentary behavior, diet quality, wellbeing), change over the three waves of data collection. A growth model will be used because it accommodates; 1) modelling of change over time; and, 2) simultaneous modelling of change occurring within an LGA over time, and between LGAs over time.

### Research plan aim 2: to measure the effects of change to community-level system factors on rates of childhood obesity among Victorian children

Since HTV aims to create change at the community (LGA level) as well as the school (setting level), the proposed research plan under Aim 2 is designed to enhance understanding of how HTV has created community-level system change. It is hypothesized that:

1. HTV changes elements of local community-level systems that influence childhood obesity
2. Differential changes in local community-level systems have differential effects on the LGA policy environment, school environments, child behavior and subsequent weight status

The Foresight obesity systems map [13] represents a watershed moment for systems thinking and obesity prevention as it provides the first visual representation of the complex causes of obesity. The resulting map begins with obesity at an individual level and builds to a peripheral set of 108 variables, clustered in seven domains (physiology, individual physical activity, physical activity environment, individual psychology, social psychology, food consumption and food production) that directly or indirectly influence obesity [13]. Five of these domains (individual physical activity, physical activity environment, individual psychology, social psychology and food consumption) will be incorporated into the analysis. The domains of physiology and food production have not been included at this time as the data are not readily available or are not collected at a population level. For example, physiology includes ‘level of satiety’ and level of thermogenesis’, both of which are not easily collected en masse. Similarly the food production domain contains variables which are difficult to measure such as ‘Palatability of food offerings’ and ‘Pressure for growth and profitability’.

### Study design and sampling

In order to test the stated hypothesises, data routinely collected by the Victorian Department of Health and Human Services plus other freely available data will be used to map accepted relationships between childhood obesity and its determinants as defined by the Foresight obesity systems map at the LGA level [13]. A detailed description of the study design, sampling strategy, protocols and procedures for each of these data sources are outlined in Table 2. Table 3 provides details of the available data items that will be used in the analysis of the systems influencing obesity. The data sources contain a mixture of child and adult data. While the primary interest of this study is childhood obesity, it is obesity at an LGA level not at an individual level. Data collected only from adults such as ‘Health Literacy for Healthy Eating and Physical Activity’, 'Affective attitudes (facilitators & barriers, decisional balance) for Healthy Eating and Physical Activity’, and ‘Self-efficacy for Healthy Eating and Physical Activity’ will be used to populate parameters at the LGA level as these are seen as systems indicators. Similarly adult anthropometric data will be included in the models in order to control for the influence of adult BMI on child BMI. It would be expected that there would be a medium to strong correlation even at the LGA level between these variables. How these systems indicators plus individual level behaviors impact childhood obesity will then be tested.

**Table 2:** Detailed description of datasets to be incorporated in Aim 2

Survey	Description (Study design, sampling and study protocol)	Participants, recruitment measures and procedures	Outcome(s) of interest	Instrument/measure
Victorian Population Health Survey (VPHS) [58]	Established in 1998, the VPHS is a Victorian representative surveillance survey that collects detailed information on the health, lifestyle and wellbeing status of Victorian adults. Random digit dialling of registered telephone numbers was used in each study year (annually from 2001) with participants completing a computer assisted telephone interview (CATI). In 2008 and 2011/12, sampling occurred in all of the 79 LGAs within Victoria and represented baseline measures for HTV. A target sample of 426 interviews per LGA was achieved. Triennial LGA-level data collection among over 33,000 is envisaged for the duration of the HTV (2014 and 2017).	• 33,673 adults (≥18 years) in private dwellings recorded complete interviews between Sept 24 and Dec 16 2008 in HTC intervention and comparison communities • All residential households with land-line telephone connections were considered eligible for the study • Interviewers made up to six calls on different days of the week and times to establish contact with the household, which was controlled by a customised call algorithm • The average interview length was approximately 22 min. • The response rate was 64.9 %.	• Change in BMI score • Change in overweight and obesity prevalence • Change in fruit and vegetable intake • Change in the proportion meeting the Australian physical activity guidelines for adults [59]	Self-rated health status
				CATI (smoking, fruit and vegetable intake, alcohol consumption, levels of PA)
				Self-reported height and weight
Preventive Health Survey (PHS)	The Preventive Health Survey was undertaken in 2011/12 and is again scheduled for completion in 2016/2017. The PHS furthers the information derived from the VPHS as it is able to make comparison estimates for each intervention HTV area compared to its’ matched-control area. This is in contrast to the VPHS, which can only make estimates for intervention and comparison HTV areas combined.	• ≥9,500 adults (≥18 years) and ≥ 1800 children (5–18 years) randomly sampled from HTV intervention and comparison communities	• Change in BMI • Change in overweight and obesity prevalence • Change in the proportion of participants meeting Australia’s physical activity and sedentary behavior guidelines for children (5–12 years) [33] and young people (13–18 years) [34] • Change in the proportion of participants eating the required amount of fruit and vegetables [39]	Self-rated health status CATI (PA, SB environmental amenity, perceived barriers and facilitators to healthy eating and activity)
				Self-reported height and weight
School Activity Audits	The Healthy Together Achievement Program is currently being offered to all Victorian Primary and Secondary schools. The program began in 2011, and was developed by the Department of Education and Training and Department of Health & Human Services (Victoria). The Achievement Program is designed to support schools to become health promoting schools and aide in the attainment of state-wide benchmarks for health promotion. Through participation in the Achievement program, schools provide information on the schools’ engagement in prevention efforts.	• Primary and Secondary Schools involved in the Healthy Together Achievement Program • The schools’ Achievement Program Coordinator completes the audits via the online registration system.	• Uptake and dispersion of the Achievement Program across Victoria • Change in progression through the various stages of the Achievement Program and achievement of benchmarks among HTV and comparison communities over time.	Online School Activity audit
LGA Policy Metrics	The Victorian Department of Health & Human Services has conducted a content analysis of Municipal Public Health and Wellbeing Plans and Council Plans in relation to healthy eating and physical activity, as well as other health and wellbeing priorities, of the 79 LGAs in Victoria. Local council plans between 2009 and 2013 have been audited in line with the 4 year municipal planning cycles.	• Content analysis of municipal documents from 79 LGAs in Victoria	• Evaluation of changes in the number and type of health and wellbeing policies within HTV intervention and comparison communities	Municipal policy documents

**Table 3:** Details of the Foresight domains [59] and variables and the items that will be used to measure them

Foresight domain	Foresight variable	Foresight description	Data collection tool	Item
Physical activity environment	Accessibility to opportunities for physical exercise	Physical accessibility (distance, safety) of opportunities for physical exercise	PHS	Perceived environmental amenity
	Ambient temperature	Average environmental temperature indoors	Not available from surveys.	Ambient temperature
			Bureau Of Meteorology (BOM)	
			http://www.bom.gov.au/products/IDV65079.shtml	
	Cost of physical exercise	Financial cost of physical recreation	Not available from surveys	
			Data available from individual LGAs	
	Dominance of motorised transport	Degree to which motorised transport dominates other ways of transport	PHS	Perceived environmental amenity
			Data available from individual LGAs	
				Combined with
				Availability of public transport
	Dominance of sedentary employment	Degree to which average citizens influence one another’s choices	Data available from individual LGAs	Employment statistics
	Opportunity for team-based activity	N/A	Not available from surveys	Register of the number of sporting clubs in each LGA
			Data available from individual LGAs	
	Opportunity for unmotorised transport	Availability of facilities/infrastructure for unmotorised transport	PHS	Perceived environmental amenity
	Perceived danger in environment	N/A	Available from the Community Indicators Victoria website by LGA	Perceptions of safety
	Reliance of labor-saving devices	Reliance on labor-saving devices for daily chores	Not available from surveys	
	Safety of unmotorised transport	Level of risk for harm by engaging in non-motorised transport	PHS	Perceived environmental amenity
	Social depreciation of labor	Degree to which manual labor is negatively valued in a given socio-cultural group	Not available from surveys	Employment demographics
			Can be inferred from the local demographics available on the Local Government websites	
	Sociocultural valuation of physical activity	Degree to which physical activity is positively valued in a given socio-cultural group	PHS	Social environment (social norms) for HEPA
	Walkability of living environment		PHS	Perceived environmental amenity
			Available from the Community Indicators Victoria website by LGA	
				School walkability
Individual physical activity	Degree of innate activity in childhood	Degree to which physical activity is part of typical childhood behavior	PHS and WHOCC surveys	Level of physical activity
	Degree of physical education	Degree to which people have learned to use their body (for labor, leisure and transport)	VPHS	Frequency and amount of vigorous physical activity in past week
			PHS	
				Physical Activity and sedentary
	Functional fitness	Level of physical fitness to perform daily tasks	VPHS	Self-reported health status
			WHOCC	
				Paediatric Quality of Life Inventory (PedsQL) [40]
	Learned activity patterns in early childhood	Degree of activity experienced by the foetus, newborn and child in early life through parental physical activity	Not available from surveys	
	Level of domestic activity	Level of physical activity exhibited in the domestic arena	VPHS	Physical activity at work
	Level of occupational activity	Level of physical activity associated to professional duties	VPHS	Physical activity at work
	Level of recreational activity	Degree to which people engage in physical activity for recreation	VPHS	Frequency and amount of vigorous physical activity in past week
			PHS	
				Physical Activity and sedentary
	Level of transport activity	Level of physical activity associated to transport	Not available from surveys	
			Available from the Community Indicators Victoria website by LGA for Melbourne metropolitan area only	
	Non-volitional activity (NEAT)	extent to which people engage in non-volitional activity (twitching etc.)	Not available from surveys	
	Parental modelling of activity	Degree to which parents act as a role model in physical activity related behavioral patterns	PHS	Parent physical activity
				Child physical activity
	Physical activity	Level of physical activity people engage in	VPHS	Frequency and amount of vigorous physical activity in past week
			PHS	
				Physical Activity and sedentary
Social psychology	Acculturation	Degree to which a (dominant) culture is assimilated	VPHS	Country of birth
				Main language spoken at home
				Country of birth of mother
				Country of birth of father
	Availability of passive entertainment options	Availability of recreational options that involve only limited physical exercise (tv, computer games)	PHS	Sedentary behavior items
	Children’s control of diet	Degree to which children exert influence on dietary choices in a family	Not available from surveys	
	Conceptualisation of obesity as a disease	Degree to which people consider obesity to be an abnormal deviation from the healthy norm	Not available from surveys	
	Education	N/A	VPHS & PHS	Demographics
	Exposure to food advertising	N/A	Not available from surveys	Level of exposure to food advertising
			Available from the literature	
	Importance of ideal body-size image	Degree to which there is a dominant image of an ideal body size in a society	Not available from surveys	
	Media availability	Availability of media across formats	Not available from surveys	Data on availability of all types of media
			The Victorian Government digital innovation review Part B: The digital readiness of Victorian citizens	
	Media consumption	Degree to which people make use of the media offerings	Not available from surveys	Data on use of all types of media
			The Victorian Government digital innovation review Part B: The digital readiness of Victorian citizens	
	Parental control	Level of control exerted by parents on children’s choices	Not available from surveys	
	Peer pressure	Degree to which average citizens influence one another’s choices	PHS	Social environment (social norms) for Healthy Eating and Physical Activity
	Perceived lack of time	By all citizens, particularly those engaged in economic activity	PHS	Instrumental beliefs (facilitators & barriers, decisional balance) for Healthy Eating and Physical Activity
	Smoking cessation	Number of people quitting smoking	VPHS	Number of people smoking from one survey to the next
	Social acceptability of fatness	N/A	VPHS & PHS	Height and weight; BMI heterogeneity between LGAs
	Sociocultural valuation of food	Degree to which food is positively valued within a given socio-cultural group	PHS	Instrumental
	TV watching	Time spent watching tv	PHS	Sedentary behavior
Individual psychology	Demand for indulgence/compensation	Strength of demand for psychological release after stress or effort	Not available from surveys	
	Desire to resolve tension	Desire to resolve psychological conflict between what people desire and what they need to stay healthy	PHS	Affective attitudes (facilitators & barriers, decisional balance) for Healthy Eating and Physical Activity
				Combined with
				Behavioral intentions (desire to change behavior) for HEPA
				And Daily vegetable consumption
				Daily fruit consumption
				Physical Activity Sedentary behavior
				beliefs (facilitators & barriers, decisional balance) for Healthy Eating and Physical Activity
	Food literacy	Degree to which people are able to assess nutritional quality and provenance	PHS	Health Literacy for Healthy Eating and Physical Activity
	Individualism	Weakness of social fabric	PHS	Social environment (social norms) for Healthy Eating and Physical Activity
			vailable from the Community Indicators Victoria website by LGA	
				Level of social support
	Perceived inconsistency of science-based messages	Degree to which there is incompatibility between scientific assessments on food related issues which (are perceived) to be similar	PHS	Health Literacy for Healthy Eating and Physical Activity
	Psychological ambivalence	Degree to which people experience a psychological conflict between what people desire (e.g., fatty, sweet foods) and the need to stay healthy	PHS	Affective attitudes (facilitators & barriers, decisional balance) for Healthy Eating and Physical Activity
	Self esteem	Sense of purpose and self-confidence of individuals	PHS	Self-efficacy for Healthy Eating and Physical Activity
	Stress	Perceived level of stress by individuals	VPHS	Psychological distress (Kessler 10 Psychological Distress Scale)
	Use of medicines	N/A	VPHS	Diabetes status
				Type of diabetes
				Age first diagnosed with diabetes
				Type of healthcare received in past year
Food consumption	Alcohol consumption	N/A	VPHS	Whether had an alcoholic drink of any kind in previous
				12 months
				Frequency of having an alcoholic drink of any kind
				Amount of standard drinks consumed when drinking
				Level of frequency of high-risk drinking
	Convenience of food offerings	The degree to which food offerings cater to the desire for convenience	PHS	Food accessibility
	Demand for convenience	Consumer demand for convenient (time/effort saving) food offerings	PHS	Instrumental beliefs (facilitators & barriers, decisional balance) for Healthy Eating and Physical Activity
	De-skilling	The degree to which individuals are not able anymore to engage independently in routine tasks for daily living (such as cooking)	VPHS	Self-reported health status
				Combined with
				Number and type of chronic diseases
	Energy-density of food offerings	Number of calories per unit food weight	VPHS	Calculated from: Daily vegetable consumption
			PHS	
				Daily fruit consumption
				Milk consumption
				Water consumption
				Consumption of sugar-sweetened soft drinks
				Daily vegetable consumption
				Daily fruit consumption
	Fibre content of Food & Drink	N/A	VPHS	Daily vegetable consumption
			PHS	
				Daily fruit consumption
				Milk consumption
				Water consumption
				Consumption of sugar-sweetened soft drinks
				Daily vegetable consumption
				Daily fruit consumption
	Food abundance	The aggregate amount of food (volume) that is at any moment in time available in UK (AU) society	Not available from surveys	The amount of food available per person in Australia
			Australian Institute of Health and Welfare 2012. Australia’s food & nutrition 2012. Cat. no. PHE 163. Canberra: AIHW.	
	Food exposure	The number of food cues individuals are confronted with on a daily basis	Not available from surveys	
			Available from the literature	
	Food variety	The number of different food products (natural and processed) available at any moment in time	Not available from surveys	Categories of the amount of food available per person in Australia
			Australian Institute of Health and Welfare 2012. Australia’s food & nutrition 2012. Cat. no. PHE 163. Canberra: AIHW.	
	Force of dietary habits	The degree to which behavioral patterns related to food intake are dictated by routine and habit	PHS	Habit strength for Healthy Eating and Physical Activity
	Nutritional quality of Food & Drink	N/A	VPHS	Daily vegetable consumption
			PHS	
				Daily fruit consumption
				Milk consumption
				Water consumption
				Consumption of sugar-sweetened soft drinks
				Daily vegetable consumption
				Daily fruit consumption
	Palatability of food offerings	N/A	Not available from surveys	
	Portion size		Not available from surveys	
	Rate of eating	Time-span devoted to consuming a meal	Not available from surveys	
	Tendency to graze	Tendency to eat outside fixed meal times	Not available from surveys	

### Data analysis

The Foresight map will be used as a basis to model relationships within and between the domains so as to demonstrate which elements of the system are changing, the strength of these changes, the elements that are resisting change and the implications of these relationships for childhood obesity.

The outlined datasets contain >75 % of the 65 variables within five domains of the Foresight map (individual physical activity, physical activity environment, individual psychology, social psychology and food consumption) and various techniques will be used to test the hypotheses. For hypothesis 1: HTV changes elements of local community-level systems that influence childhood obesity, a series of mixed effects linear regression analyses will be used. Prior to the analyses, a confirmatory factor analysis will be utilised to reduce the data within each of the Foresight domains so a factor score is obtained. Each of the factors will then be used as the dependent variable with the independent variable being study condition (HTV or non-HTV) interacting with time adjusted for hypothesised covariates. The resulting predictions will be plotted and compared to the changes in childhood obesity from Aim 1. Positive changes in each of the domains in HTC compared to non-HTC combined with positive changes in childhood obesity over the same time period would provide support for Hypothesis 1.

For hypothesis 2: Differential changes in local community-level systems have differential effects on the LGA policy environment, school environments, child behavior and subsequent weight status, a series of multi-level (individual and LGA) structural equation models will be used. The main area of interest will be the LGA level as it is the heterogeneity at this level that is important in testing hypothesis two. The first model will test the influence of each of the domains on one another plus policies within LGAs. The second will substitute school environments as the dependent variable, the third child behavior and the fourth child weight status. Child weight status will be calculated using the self-reported data from the PHS. These data will be used as they have corresponding adult data that can be used in calculating the systems-level exposures. Sensitivity analyses will be undertaken in order to calculate the differences in child weight status between the measured and self-report groups. Depending on findings, weightings may be used to correct the difference.

Using this approach, the impact of changes within each of the five domains can be quantified and the relative contribution of each domain to LGA policy environment (continuous score of number of obesity related policies calculated from the policy audits), school environments (continuous score calculated from the School Environmental Audits), child behaviour (proportion of children meeting the PA and sedentary guidelines and proportion meeting the fruit and vegetable consumption guidelines) and subsequent weight status (proportion of children with overweight and obesity) assessed over time. Model fit analyses (e.g., Aikake’s and Bayesian Information Criteria) will be used to determine the significance of the impact of the each of the domains of the system, individually and in total, both cross-sectionally and over time.

## Discussion and conclusion

This Healthy Together Victorian and Childhood Obesity research plan outlines a detailed approach for measuring both individual and community changes in obesity and associated risk factors for a population-level community-based intervention. HTV represents a pioneering application of systems thinking to the primary prevention of chronic disease among children and adults in Victoria, Australia. Employment of an opt-out consent process represents an Australian first for behavioral studies of this type. Other projects suggest an opt-out consent process will result in a response rate in excess of 90 % [51]. In addition, the plan will incorporate these data with broader system measures to better understand system interventions

The employment of a cluster RCT of entire communities to determine effectiveness of the HTV is challenging, although the heterogeneity of systems changes and outcomes between communities will be able to be captured and examined under the second research plan. Additionally, the randomisation of communities in HTV is another strength over other large-scale ‘whole of community’ obesity/chronic-disease prevention interventions; as other community interventions have instead used a mixture of quasi-experimental research designs or non-randomized comparators [22, 52–54]. Intervention researchers are faced with complex decisions when designing, implementing and evaluating complex interventions to ensure that they that are both acceptable and practical to the people and communities they effect and also to the funding and governing bodies overseeing the projects. The measures for Aim 1 have been selected based on their psychometric properties (reliability and validity) among children and adolescents, but are subject to measurement errors including non-response bias, respondent bias such as social desirability, self-report, as well as respondent memory lapses and coding errors [35]. Although these can never be eradicated, detailed protocols have been developed alongside compulsory training and strict supervision structures for data collection and management to reduce their potential influence. Social desirability is less of an issue in young children than in adults. Regardless of bias, most self-report studies find that people do not meet the recommended levels of fruit and vegetable consumption. The issue of non-response bias is minimized through the employment of opt-out consent and through the use of electronic data collection to collect data as participants are prompted to complete all question items. A further limitation was that the original HTV study design included areas of more disadvantage and higher prevalence of chronic disease risk factors. This bias may result in results skewed towards higher prevalence of childhood obesity and so may not be generalizable to the wider population.

There is definitive need for large-scale, population-based approaches to prevent obesity/chronic disease globally. Creating a system intervention at this scale is unprecedented and to date methods to understand the possible effectiveness of such an approach have been theoretical or relied on simulation modelling. This protocol represents the first attempt to understand changes in obesity and associated risk factors through HTV using a solution-oriented research paradigm [55] that is based on strong trial study design, but also introduces flexibility to use routine government data in order to understand systems changes.
